# Supplementary material for: Recombination Between Bubaline Alphaherpesvirus 1 and Bovine Alphaherpesvirus 1 as a Possible Origin of Bovine Alphaherpesvirus 5
Source: Viruses. 2025 Jan 30;17(2):198. doi: 10.3390/v17020198 (PMC11862017; doi:10.3390/v17020198)
Supplement: Supplementary file 1 [file viruses-17-00198-s001.zip › Table S1.pdf]

Table S1. List of genome sequences used for phylogenetic analysis. Name of strain/isolate, country of origin and GenBank accession number of sequences used in this study.

Genbank accession	Virus	Abbreviation	Strain(s) or isolate(s)	Country(ies)
MG407786	Bovine alphaherpesvirus 1 subtype 1	BoAHV1.1	MN12	USA
MG407791	Bovine alphaherpesvirus 1 subtype 1	BoAHV1.1	PA2	USA
MG407792	Bovine alphaherpesvirus 1 subtype 1	BoAHV1.1	PA3	USA
MG407788	Bovine alphaherpesvirus 1 subtype 1	BoAHV1.1	MN14	USA
MG407789	Bovine alphaherpesvirus 1 subtype 1	BoAHV1.1	MN15	USA
MG407787	Bovine alphaherpesvirus 1 subtype 1	BoAHV1.1	MN13	USA
MG407785	Bovine alphaherpesvirus 1 subtype 1	BoAHV1.1	MN11	USA
NC_063268	Bovine alphaherpesvirus 1 subtype 1	BoAHV1.1	NVSL Cooper	USA
MG407778	Bovine alphaherpesvirus 1 subtype 1	BoAHV1.1	MN4	USA
MG407775	Bovine alphaherpesvirus 1 subtype 1	BoAHV1.1	MN1	USA
MG407783	Bovine alphaherpesvirus 1 subtype 1	BoAHV1.1	MN9	USA
MG407784	Bovine alphaherpesvirus 1 subtype 1	BoAHV1.1	MN10	USA
MG407779	Bovine alphaherpesvirus 1 subtype 1	BoAHV1.1	MN5	USA
MG407777	Bovine alphaherpesvirus 1 subtype 1	BoAHV1.1	MN3	USA
MG407780	Bovine alphaherpesvirus 1 subtype 1	BoAHV1.1	MN6	USA
MG407782	Bovine alphaherpesvirus 1 subtype 1	BoAHV1.1	MN8	USA
MG407790	Bovine alphaherpesvirus 1 subtype 1	BoAHV1.1	PA1	USA
MG407781	Bovine alphaherpesvirus 1 subtype 1	BoAHV1.1	MN7	USA
AJ004801	Bovine alphaherpesvirus 1 subtype 1	BoAHV1.1	K22, Cooper, P8-2, 34 and Jura	USA, Canada, Switzerland
KY215944	Bovine alphaherpesvirus 1 subtype 1	BoAHV1.1	216 II	India
MH791338	Bovine alphaherpesvirus 1 subtype 1	BoAHV1.1	C44	USA
KU198480	Bovine alphaherpesvirus 1 subtype 1	BoAHV1.1	Cooper	USA
MH791339	Bovine alphaherpesvirus 1 subtype 1	BoAHV1.1	C45	USA
MH791340	Bovine alphaherpesvirus 1 subtype 1	BoAHV1.1	C46	USA
MH724210	Bovine alphaherpesvirus 1 subtype 1	BoAHV1.1	Nasalgen IP MLV vaccine	USA
MH724202	Bovine alphaherpesvirus 1 subtype 1	BoAHV1.1	Arsenal IBR MLV vaccine	USA
MH724203	Bovine alphaherpesvirus 1 subtype 1	BoAHV1.1	Titanium IBR MLV vaccine	USA

MH724204	Bovine alphaherpesvirus 1 subtype 1	BoAHV1.1	Express 1 IBR MLV vaccine	USA
MH724205	Bovine alphaherpesvirus 1 subtype 1	BoAHV1.1	Pyramid IBR MLV vaccine	USA
MH724206	Bovine alphaherpesvirus 1 subtype 1	BoAHV1.1	Vista IBR MLV vaccine	USA
MH751900	Bovine alphaherpesvirus 1 subtype 1	BoAHV1.1	C29	USA
MH724207	Bovine alphaherpesvirus 1 subtype 1	BoAHV1.1	BoviShield IBR MLV vaccine	USA
MH724208	Bovine alphaherpesvirus 1 subtype 1	BoAHV1.1	BoviShield Gold FP5 MLV	USA
MH751898	Bovine alphaherpesvirus 1 subtype 1	BoAHV1.1	C18	USA
MH751899	Bovine alphaherpesvirus 1 subtype 1	BoAHV1.1	C26	USA
MH751901	Bovine alphaherpesvirus 1 subtype 1	BoAHV1.1	C33	USA
MF421714	Bovine alphaherpesvirus 1 subtype 1	BoAHV1.1	Los Angeles	USA
MH724209	Bovine alphaherpesvirus 1 subtype 1	BoAHV1.1	TSV-2 Nasal MLV vaccine	USA
MH791336	Bovine alphaherpesvirus 1 subtype 1	BoAHV1.1	C42	USA
MH791341	Bovine alphaherpesvirus 1 subtype 1	BoAHV1.1	C47	USA
MH791337	Bovine alphaherpesvirus 1 subtype 1	BoAHV1.1	C43	USA
MH598937	Bovine alphaherpesvirus 1 subtype 1	BoAHV1.1	C35 1839-9847	USA
MH598938	Bovine alphaherpesvirus 1 subtype 1	BoAHV1.1	C28 55771	USA
MH598936	Bovine alphaherpesvirus 1 subtype 1	BoAHV1.1	C14 CSU 034-10640	USA
OR211605	Bovine alphaherpesvirus 1 subtype 1	BoAHV-1	16453/07 TN	Italy
PP696974	Bovine alphaherpesvirus 1 subtype 1	BoAHV-1.1	005295	Ireland
KM258880	Bovine alphaherpesvirus 1 subtype 1.2	BoAHV1.2b	K22	USA
OP035381	Bovine alphaherpesvirus 1 subtype 1.2	BoAHV1.2b	BHV SHJS	China
KM258881	Bovine alphaherpesvirus 1 subtype 1.2	BoAHV1.2b	B589	Australia
KM258882	Bovine alphaherpesvirus 1 subtype 1.2	BoAHV1.2b	SM023	USA
KM258883	Bovine alphaherpesvirus 1 subtype 1.2	BoAHV1.2b	SP1777	USA
MG407776	Bovine alphaherpesvirus 1 subtype 1.2	BoAHV1.2b	MN2	USA
MW829288	Bovine alphaherpesvirus 5 subtype b	BoAHV5b	A663	Argentina
MZ364295	Bovine alphaherpesvirus 5 subtype b	BoAHV5b	166/84	Argentina
MZ420492	Bovine alphaherpesvirus 5 subtype b	BoAHV5b	674/10	Argentina

NC_005261	Bovine alphaherpesvirus 5 subtype a	BoAHV5a	SV507/99	Brazil
KY559403	Bovine alphaherpesvirus 5 subtype c	BoAHV5c	P160/96	Brazil
KY549446	Bovine alphaherpesvirus 5 subtype c	BoAHV5c	ISO97/45	Brazil
PP897810.1	Bovine alphaherpesvirus 5	BoAHV5	BoHV-5/Cattle/India/2018/Bhilwara	India
OQ669137	Bubaline alphaherpesvirus 1	BuAHV1	HeiferVagina-S102_1	India
OQ669139	Bubaline alphaherpesvirus 1	BuAHV1	Docile-S101_1	India
OQ669138	Bubaline alphaherpesvirus 1	BuAHV1	Milk-S104_1	India
NC_043054	Bubaline alphaherpesvirus 1	BuAHV1	b6	Australia
OQ442798	Bubaline alphaherpesvirus 1	BuAHV1	IZSM_BuHV1_01	Italy
OQ608621	Bubaline alphaherpesvirus 1	BuAHV1	S103	India
